# The Utility of Serial Lipid Measurements as a Potential Predictor of Sepsis Outcome: A Prospective Observational Study in a Tertiary Care Hospital

**DOI:** 10.2478/jccm-2024-0015

**Published:** 2024-04-30

**Authors:** Afrah Abdul Malick, Jeyakumar Manavalan, Viveka Murugiah, Manikandan Bose, Hariharan Alexander, Suganthy Kanakasekaran

**Affiliations:** Velammal Medical College Hospitals and Research Institute, Madurai, Tamil Nadu, India; Sri Manakula Vinayagar medical college and Hospital, Puducherry, India

**Keywords:** sepsis, procalcitonin, HDL-cholesterol, triglycerides, APACHE II score

## Abstract

**Background and aim:**

Sepsis is the major cause of morbidity and mortality for patients admitted to an intensive care unit worldwide. Currently, procalcitonin (PCT) is a widely used prognostic marker for sepsis. The high cost of estimating Procalcitonin limits its utility in all health facilities. Lipid profile, being a frequently done routine investigation, is studied in sepsis patients to predict the prognosis of sepsis. This study was aimed to assess the association between lipid profile parameters, procalcitonin and clinical outcomes in patients with sepsis.

**Materials and methods:**

It is a prospective observational study conducted in a tertiary care hospital in the Department of Biochemistry in collaboration with the Intensive Care Unit (ICU). We included 80 sepsis patients from medical and surgical ICUs. Among them, 59 (74%) survived and 21 (26%) expired. Serum lipid profile, procalcitonin and variables required for APACHE II score are measured at two intervals, one during admission and on day 5. All the parameters were compared between the survivors and the non-survivors.

**Results:**

Serum PCT levels were reduced on day 5 [3.32 (1.27–11.86)] compared to day 0 [13.42 (5.77–33.18)] in survivors. In survivors, Total Cholesterol, LDL-C and Non-HDL-C were significantly elevated on day 5 compared to day 0. In non-survivors, HDL-C significantly decreased on day 5. Between survivors and non-survivors, HDL-C significantly decreased on day 5 (23.88 ± 10.19 vs 16.67 ± 8.27 mg/dl). A Negative correlation was observed between HDL-C & PCT.

**Conclusion:**

Serum Lipid profile levels, namely Total cholesterol, HDL-C and LDL-C, have possible associations with the severity of sepsis. HDL-C have a negative association with the clinical scoring system in sepsis patients. Overall, the findings from our study suggest that lipid profile parameters have possible implications in predicting the outcome of patients with sepsis.

## Introduction

Sepsis is one of the most common causes of hospital admission worldwide and often requires an aggressive management protocol. Despite the advanced approach in management, sepsis accounts for the major cause of morbidity and mortality for patients admitted to an intensive care unit [1]. A prospective study conducted in a tertiary care hospital in India observed an ICU mortality of 56% and 28-day mortality of 62.8% [2].

Sepsis can be defined as life-threatening organ dysfunction caused by a dysregulated host response to infection [3]. Organ dysfunction can be identified clinically by an acute change in two or more SOFA (Sequential Organ Failure Assessment) points, along with either suspicion or evidence of infection [4]. Management protocol for sepsis patients comprises antibiotic therapy, adequate volume repletion and good glycaemic control. Despite aggressive treatment, the mortality rate due to sepsis is still high.

An early prognostic marker is essential to identify those at the highest risk for mortality to optimise treatment. Currently, Procalcitonin (PCT) is found to have better accuracy and is a widely used prognostic marker for sepsis [5]. The high cost of estimating Procalcitonin limits its utility in all healthcare facilities. Hence, it is essential to find simple, cost-effective markers to predict the progress of sepsis, which can be used in everyday practice.

The hallmark of sepsis is a dysregulated inflammatory response. Fatty acid-derived lipid mediators play a significant role in the generation and resolution of inflammation. These mediators can provide insight into the patient’s inflammatory status and immune response. Thus, lipid profiling can be used to assess the interaction between metabolic changes and immune response. These metabolic signatures can improve the chances of better prognostic and diagnostic evaluation, paving the path to personalized treatment strategies [6].

There has been evidence that serum cholesterol and lipoprotein levels change over time in septic patients. The inflammatory cascade produced by binding CD14 with lipopolysaccharides present in the outer membrane of gram-negative bacteria initiates the onset of sepsis [7]. HDL binds with the lipopolysaccharide (LPS) of gram-negative bacteria, neutralises it and helps in the clearance of Lipoteichoic Acid (LTA) produced from gram-positive bacteria [8,9].

Sequential Organ Failure Assessment (SOFA) score and Acute Physiology and Chronic Health Evaluation (APACHE) II score are prognostic models that use clinical findings and laboratory parameters to predict the severity of sepsis. APACHE II scoring system was found to be both sensitive as well as specific [10,11].

In this study, we aimed to study the association of serum lipid profile parameters, namely total cholesterol, triglycerides and high-density lipoprotein (HDL) with procalcitonin and clinical outcome by using APACHE II score in patients with sepsis.

## Materials and Methods

### Patients and Methods

This is the hospital-based prospective observational study conducted in the Department of Biochemistry after approval from the Institutional Ethics Committee. This study follows the ethical principles for medical research involving human subjects in accordance with the Declaration of Helsinki of 1975, revised in 1983. The participants were recruited from the medical and surgical ICUs from June 2022 to September 2022. Written informed consent was obtained from the patients or their legal guardians after explaining the study protocol.

### Study Population

Eighty patients from both medical and surgical intensive care units were recruited in the study after subjecting to inclusion and exclusion criteria.

Inclusion criteria: patients above 18 years of age and satisfy the criteria for diagnosing sepsis, as per international guidelines for the management of sepsis and septic shock [3].

Exclusion criteria: paediatric patients, pregnant and lactating women, patients who died within 24 hours of admission or patients who were referred to another hospital were not included in the study. Lipid profile parameters can be affected by several confounders like nutritional status, physical activity, smoking, alcohol intake, dyslipidemia, malignancy, HIV infection, autoimmune disease. Hence sepsis patients with HIV, malignancy, autoimmune diseases like systemic lupus erythematosus & rheumatoid arthritis and patients on immunosuppressive therapy, sepsis patients with dyslipidaemia and patients on statin therapy were also excluded from the study.

### Study Procedure

Detailed medical history, including demographic data, presenting complaints and past history of prolonged illness and hospitalisation, were recorded. A thorough clinical examination of the patient was done, and disease aetiology of ICU admission was recorded for each patient. All the parameters required to calculate the APACHE II score were recorded. APACHE II scores were calculated using an online calculator [12] & serum was analysed for lipid profile on day 0 & day 5. All the study participants were followed throughout the study, and the final status as discharged or deceased was obtained from the medical records department.

### Biological Sample

Under strict aseptic conditions, 5 ml of venous blood was collected in a plain serum tube from every patient on days 0 and 5 of admission. Blood was spun at 3500 rpm for 10 minutes at standard room temperature. The serum was separated, and the study parameters were estimated immediately.

### Study Parameters

All these biochemical parameters were analysed on day 0 and day 5 of admission with sepsis.

Serum total cholesterol was estimated by the CHOD-PAP (cholesterol oxidase & peroxidase) method; serum HDL – cholesterol was estimated by the selective inhibition method; serum triglyceride by GPO-TOPS (glycerol-3-phosphate oxidase & peroxidase) method. The above colorimetric assays were measured using TBA 120 FR, a fully automated chemistry analyser. Other lipid parameters like LDL & VLDL were calculated from total cholesterol, triglyceride & HDL levels using Friedewald’s formula.

Serum procalcitonin was measured using the ECLIA method using COBAS e 411, a fully automated immunoassay analyser. During analysis, laboratory standard operating procedures were followed with internal quality control materials.

### Statistical analysis

All the study variables were expressed as mean ± standard deviation or median (inter-quartile range). The parameters studied were tested for normal distribution using the Kolmogorov-Smirnov test. Differences in the serum levels of lipid parameters, procalcitonin, and APACHE II scores on day 0 and day 5 of admission were calculated using a paired t-test or Wilcoxon signed-rank test. Differences in the study parameters between survivors and non-survivors were calculated using Unpaired t-test or Mann-Whitney U test. Spearman correlation was done to evaluate the association between biochemical parameters and APACHE II score. All statistical analyses were done using SPSS version 23.0. p-value < 0.05 is considered statistically significant.

## Results

We have enrolled 95 volunteered patients from the ICU diagnosed with sepsis with various causes for 4 months. Out of which, 9 patients deceased before the 5th day of admission, and 6 patients with triglycerides more than 400 mg/dl were excluded from the study. A total of 80 patients were included in the study based on the inclusion criteria and follow-up details. Out of 80 patients, 59 (74%) survived, and 21 (26%) were non-survivors. Coronary artery disease, respiratory diseases and trauma are the three leading causes of sepsis in the study participants (Table 1).

**Table 1.** Baseline characteristics of the study participants

**Parameters**	**Number of Patients (n = 80)**
Total number of cases	80
Male: Female	56:24
	Males (70%); Females (30%)
Discharged (Survivors)	59 (74%)
Deceased (Non-Survivors)	21 (26%)

**Parameters**	**Median (Interquartile Range)**
Age (in years)	57 (45–66)
Number of Days in ICU	13 (8–17)

Diagnosis	**Number of Patients (%)**
CAD	16 (20%)
Necrotizing Fasciitis	7 (9%)
GIT & Liver disease	2 (2.5%)
Renal disease (Pyelonephritis)	8 (10%)
Trauma	12 (15%)
DM Complications (No statin therapy)	2 (2.5%)
Respiratory diseases	12 (15%)
Others	21 (26%)

**Fig. 1.**
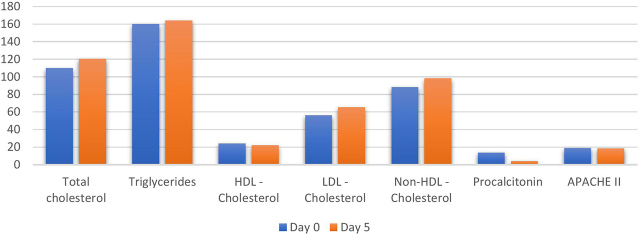
DIfference in biochemical parameters on day 0 and day 5 of admission - both survivors and non survivors

Procalcitonin, HDL – C and APACHE II Score were significantly decreased on day 5 compared to day 0, while total cholesterol, LDL – C and non-HDL cholesterol were significantly elevated on day 5 compared to day 0 in both survivors and non-survivors (Table 2 and Figure 1). Meanwhile, TGL values were elevated but not statistically significant (p = 0.13).

**Table 2.** Difference in biochemical parameters on day 0 & day 5 of admission – paired t-test / Wilcoxon signed-rank test – both survivors & non-survivors

**Parameters**	**Day 0**	**Day 5**	**p-value**
Total cholesterol (mg/dL)	109.83±38.17	120.16±39.39	0.024^*^
Triglycerides (mg/dL)	157 (104–208)	164 (114–225)	0.135
HDL - Cholesterol (mg/dL)	24.60±12.19	21.99±10.19	0.032^*^
LDL - Cholesterol (mg/dL)	55.51±30.81	63.76±32.40	0.034^*^
Non-HDL - Cholesterol (mg/dL)	87.99±35.96	98.18±38.62	0.020^*^
Procalcitonin (ng/mL)	13.7 (5.04–38.21)	4 (1.45–13.43)	0^*^
APACHE II	17 (14–25)	17 (13–23)	0.001^*^

* p values < 0.05 are considered statistically significant.

In survivors, procalcitonin was significantly decreased on day 5 compared to day 0, while total cholesterol, LDL – C and non-HDL cholesterol were significantly elevated on day 5 compared to day 0 (Table-3).

**Table 3.** Difference in biochemical parameters on day 0 & day 5 of admission – paired t-test / Wilcoxon signed-rank test – in survivors

**Parameters**	**Day 0**	**Day 5**	**p-value**
Total cholesterol (mg/dL)	108.29±37.29	123.59±39.30	0.005^*^
Triglycerides (mg/dL)	159 (107–212)	148 (115–230)	0.339
HDL - Cholesterol (mg/dL)	25.25±12.41	23.88±10.19	0.315
LDL - Cholesterol (mg/dL)	53.27±30.05	65.97±33.25	0.008^*^
Non-HDL - Cholesterol (mg/dL)	86.78±35.16	99.71±39.27	0.014^*^
Procalcitonin (ng/mL)	13.42 (5.77–33.18)	3.32 (1.27–11.86)	0^*^
APACHE II	16 (12–21)	16 (14–20)	0.183

* p values < 0.05 are considered statistically significant.

In non - survivors, HDL–C was significantly decreased on day 5 compared to day 0, while the APACHE II score was elevated on day 5 compared to day 0 (Table 4). Serum triglyceride levels were elevated but not statistically significant.

**Table 4.** Difference in biochemical parameters on day 0 & day 5 of – paired t-test / Wilcoxon signed-rank test – in non-survivors

**Parameters**	**Day 0**	**Day 5**	**p-value**
Total cholesterol (mg/dL)	114.14±41.15	110±38.95	0.654
	111(70–149)	115 (76–143)	
Triglycerides (mg/dL)	152.38±66.18	178.33±93.79	0.139
HDL - Cholesterol (mg/dL)	22.76±11.67	16.67±8.27	0.019^*^
LDL - Cholesterol (mg/dL)	61.81±32.76	57.57±29.77	0.499
Non-HDL - Cholesterol (mg/dL)	91.38±38.82	93.86±37.32	0.753
Procalcitonin (ng/mL)	15.53 (4.4–41.75)	7.0 (2.4–17.07)	0.11
APACHE II	23 (17.5–26.0)	28 (24–30)	0^*^

* p values < 0.05 are considered statistically significant.

When comparing the lipid profile between survivors & non-survivors, no significant difference was observed on day 0. On day 5, HDL-C showed a significant decrease in non-survivors. APACHE II score was significantly increased in non-survivors on both day 0 & day 5 (Table 5).

**Table 5.** Difference in biochemical parameters between survivors & non-survivors on day 5 of admission – unpaired t-test / Mann Whitney U test

**Parameters**	**Survivors**	**Non-Survivors**	**p-value**
Total cholesterol (mg/dL)	123.59±39.304	110.52±38.947	0.193
Triglycerides (mg/dL)	148 (115–230)	176 (101–223)	0.810
HDL - Cholesterol (mg/dL)	23.88±10.190	16.67±8.272	0.005^*^
LDL - Cholesterol (mg/dL)	65.97±33.254	57.57±29.772	0.311
Non-HDL - Cholesterol (mg/dL)	99.71±29.275	93.86±37.317	0.554
Procalcitonin (ng/mL)	3.32 (1.27–11.86)	7.0 (2.4–17.07)	0.170
APACHE II	16 (14–20)	28 (24–30)	0^*^

* p values < 0.05 are considered statistically significant

A correlation study was done using Spearman correlation between lipid profile and procalcitonin & APACHE II among survivors & non-survivors on day 0 & day 5.

Total cholesterol, LDL – C & non-HDL – C showed a negative correlation with Procalcitonin on day 0. Meanwhile, HDL-C showed a negative correlation with PCT on day 5 (Table 6).

## Discussion

Sepsis is a life-threatening disorder characterized by the systemic inflammatory response to microbial invasion. As per WHO, in 2017, the major contributors to sepsis and its related mortality across all ages were diarrheal diseases and respiratory infections. Since non-communicable diseases are increasing, nearly half of sepsis-related deaths were due to chronic illness [13]. Patients affected with infection, severe injury or serious noncommunicable disease can progress to sepsis. However, older persons, hospitalised patients, and patients in intensive care units are at higher risk of developing sepsis [14]. In our study, sepsis was higher in patients with underlying systemic illness. Causative organisms for healthcare-associated infections are often multi-drug resistant to drugs, and antimicrobial resistance contributes majorly to clinical unresponsiveness to treatment and rapid evolution to sepsis and septic shock [15]. In our study, the Gram-negative bacteria are the most frequent cause of sepsis. The bacteria identified in the blood culture are *Klebsiella pneumoniae, Escherichia coli, Pseudomonas aeruginosa, Acinetobacter baumannii, Enterococcus* and *Staphylococcus aureus*. Early diagnosis and appropriate antibiotic administration are crucial for saving lives. Despite blood culture being considered as gold standard investigation for septicaemia, delayed diagnosis of sepsis is the main drawback. Early recognition of sepsis prevents the transition into septic shock, which is associated with a 40% mortality rate [16]. PCT is a better biomarker, helpful for early detection of sepsis and can be used to track the disease course and guide in therapeutic intervention [17]. In this study, PCT levels were compared among survivors and non-survivors at two-time points, day 0 and day 5, of ICU admission with sepsis. The results showed that there was a significant reduction in serum PCT levels on day 5 compared to day 0 among survivors. This reduction in PCT levels might be explained by appropriate antibiotic management in those patients with sepsis. However, there is no significant reduction among non-survivors, which can explain the poor prognosis even after antibiotic therapy. According to Dahaba AA et al. [18], procalcitonin levels in early sepsis are significantly lower among survivors than non-survivors. In our study, even though there is a reduction in PCT levels among survivors compared to non-survivors, the difference is not significant. However, the APACHE II score was significantly higher in non-survivors on both day 0 and day 5 compared to survivors. According to Brunkhorst FM et al. [19] and Iram Yunus et al. [20], PCT could be used as a complementary comparator with severity scoring systems for the prediction of survival outcomes. The correlation of PCT with the APACHE II severity scoring system in sepsis patients showed a weakly positive correlation. We have observed in the current study that the change in PCT on admission and at the end of the observation period significantly indicated the disease severity in survivors.

The high cost factor and unavailability in primary care limited the use of PCT in all healthcare systems. Fatty acid-derived lipid mediators play a significant role in the generation and resolution of inflammation. These mediators can provide insight into the patient’s inflammatory status and immune response. Thus, lipid profiling can be used to assess the interaction between metabolic changes and immune response. These metabolic signatures can improve the chances of better prognostic and diagnostic evaluation, paving the path to personalized treatment strategies [6]. Lipid profile, being a frequently done routine investigation, is studied in sepsis patients to predict the prognosis of sepsis. This study was conducted to assess the pattern of lipid profile parameters in patients with sepsis and the usefulness of lipid profile as a severity and mortality indicator in patients with sepsis. We have estimated lipid profiles in patients with sepsis on day 0 and day 5 among survivors and non-survivors. We observed statistically significant higher total cholesterol levels on day 5 of treatment compared to the day of admission, which is consistent with the previous findings by Doaa Rashwan et al. [21]. Our study observed elevated triglyceride levels between survivors and non-survivors, but not statistically significant. Sunanya P et al. and Cetinkaya et al. observed elevated triglyceride levels in patients with septic shock [22,23].

Cholesterol metabolism is related to inflammation by the influence of pro-inflammatory cytokines. HDL plays a significant role in innate and adaptive immunity. Previous studies state that there is a dramatic decrease of HDL in Sepsis due to marked consumption of HDL particles by bacterial endotoxins [24].

Morin EE et al. and Chien JY et al. have observed the association between low HDL levels and the severity of sepsis [25,26]. In our study, HDL – C showed a significant decrease on day 5 compared to day 0 in non-survivors and showed a negative correlation with APACHE II in both survivors and non-survivors. Like HDL, LDL plays a key role in neutralizing endotoxins, such as Lipopolysaccharide (LPS). Maile MD et al. observed in 2020 [27] that higher levels of LDL were associated with low mortality in sepsis patients. In our study, LDL–C was found to be significantly higher on day 5 compared to day 0 in survivors. But LDL – C was lower in non-survivors on day 5 compared to day 0, although it is not statistically significant. In addition to this, Baseline LDL-C values were found to correlate with procalcitonin in non–survivors. In our study, non-HDL-cholesterol showed a negative correlation with Procalcitonin among sepsis patients in accordance with the observation by Chang L et al. [28].

### Limitations

Being a single-centre, time-bound study, there are a few limitations. Non-uniformity in the sample size of survivors and non-survivors is the major limitation of our study. LDL–C was calculated using Friedewald’s formula, which may underestimate LDL values in patients with elevated triglyceride levels. Lipid profile was measured only on baseline (day 0) & day 5 of onset of sepsis. One or more measurements of lipid profile, including at the time of recovery, may provide more valuable information about the role of lipoproteins in sepsis patients. The nutritional status of the study participants has not been taken into consideration, which can be a potential confounder for the study results. Hence, large-scale studies are needed to confirm the role of lipids and lipoproteins in predicting outcomes of patients with sepsis.

## Conclusion

As a standalone value, procalcitonin may not be used to assess prognosis in non-survivors, whereas serial PCT measurements can be used to monitor the prognosis of sepsis in survivors. Serum lipid profile levels, namely Total cholesterol, HDL – C and LDL – C, have possible associations with the severity of sepsis. HDL–C has a negative association with the clinical scoring system in sepsis patients. Overall, the findings from our study suggest that lipid profile parameters have possible implications on the outcome of patients with sepsis.

## Highlights

As a standalone value, Procalcitonin may not be a reliable prognostic marker for sepsis
Serum Lipid profile parameters have possible implications on the outcome of patients with sepsis
Serum HDL-Cholesterol values have an inverse association with the clinical severity scoring system in sepsis

## Abbreviations

PCT: Procalcitonin
HDL: C – High-Density Lipoprotein
LDL: C – Low-Density Lipoprotein
TGL: Triglycerides
APACHE II: Acute Physiology and Chronic Health Evaluation II
LPS: Lipopolysaccharide
ECLIA: Electrochemiluminescence immunoassay
SOFA: Sequential Organ Failure Assessment score
GIT: Gastro-Intestinal Tract
DM: Diabetes Mellitus
